# Prediction and Verification of the Major Ingredients and Molecular Targets of *Tripterygii Radix* Against Rheumatoid Arthritis

**DOI:** 10.3389/fphar.2021.639382

**Published:** 2021-06-08

**Authors:** Yi Ling, Hui Xu, Nina Ren, Changming Chen, Ping Zeng, Daomin Lu, Xueming Yao, Wukai Ma

**Affiliations:** ^1^ Graduate School, Guizhou University of Traditional Chinese Medicine, Guiyang, China; ^2^ Department of Rheumatology Immunology, The Second Affiliated Hospital of Guizhou University of Traditional Chinese Medicine, Guiyang, China

**Keywords:** tripterygii radix, kaempferol, rheumatoid arthritis, TNF signaling pathway, molecular targets

## Abstract

*Tripterygii Radix* exhibits good clinical efficacy and safety in rheumatoid arthritis (RA) patients, but its effective components and mechanism of action are still unclear. The purpose of this study was to explore and verify the major ingredients and molecular targets of *Tripterygii Radix* in RA using drug-compounds-biotargets-diseases network and protein-protein interaction (PPI) network analyses. The processes and pathways were derived from Gene Ontology (GO) and Kyoto Encyclopedia of Genes and Genomes (KEGG) pathway enrichment analyses. The most important compounds and biotargets were determined based on the degree values. RA fibroblast-like synoviocytes (RA-FLS) were separated from RA patients and identified by hematoxylin and eosin (HE) staining and immunohistochemistry. The purity of RA-FLS was acquired by flow cytometry marked with CD90 or VCAM-1. RA-FLS were subjected to control, dimethyl sulfoxide (control), kaempferol, or lenalidomide treatment. Cell migration was evaluated by the transwell assay. The relative expression of biotarget proteins and cytokines was analyzed by western blotting and flow cytometry. In total, 144 chemical components were identified from *Tripterygii Radix*; kaempferol was the most active ingredient among 33 other components. Fourteen proteins were found to be affected in RA from 285 common biotargets. The tumor necrosis factor (TNF) signaling pathway was predicted to be one of the most latent treatment pathways. Migration of RA-FLS was inhibited and the expression of protein kinase B (AKT1), JUN, caspase 3 (CASP3), TNF receptor 1 and 2 (TNFR1 and TNFR2), interleukin-6 (IL-6), and TNF-α was significantly affected by kaempferol. Thus, this study confirmed kaempferol as the effective component of *Tripterygii Radix* against RA-FLS and TNF signaling pathway and its involvement in the regulation of AKT1, JUN, CASP3, TNFR1, TNFR2, IL-6, and TNF-α expression.

## Introduction

Rheumatoid arthritis (RA) is an autoimmune disease characterized with chronic synovitis and proliferation of synovial cells (Weyand and Goronzy, 2017) accompanied with the release of inflammatory factors, which destroy the bone and the cartilage and eventually lead to the loss of joint function (Clapp et al., 2016; Stanford et al., 2016). Fibroblast-like synoviocytes (FLS) constitute the main part of the synovial tissue and significantly contribute to inflammation and aggressive joint destruction in RA (Falconer et al., 2018). Therefore, it is beneficial to inhibit the migration and invasion of and RA-FLS-mediated expression of inflammatory factors in patients with RA (Ma et al., 2019).

*Tripterygii Radix* is an effective Chinese herb used to treat arthritis (Lv et al., 2015). It not only suppresses cell proliferation, migration, and invasion by apoptosis but also diminishes the expression of pro-inflammatory cytokines, pro-inflammatory mediators, adhesion molecules, and matrix metalloproteinases (Dai and Bao, 2011). Recent studies demonstrated the *T. Radix*-mediated inhibition of angiogenesis and suppression of abnormally activated innate immune response in rat kidney tissue through the modulation of the nuclear factor kappa B (NF-κB) signaling pathway (Zhang et al., 2017; Liu et al., 2018). Nevertheless, the detailed mechanisms underlying the anti-inflammatory and anti-proliferative effects of *Tripterygii Radix* on RA-FLS are unclear, given its complex ingredients*.*

**FIGURE 1:**
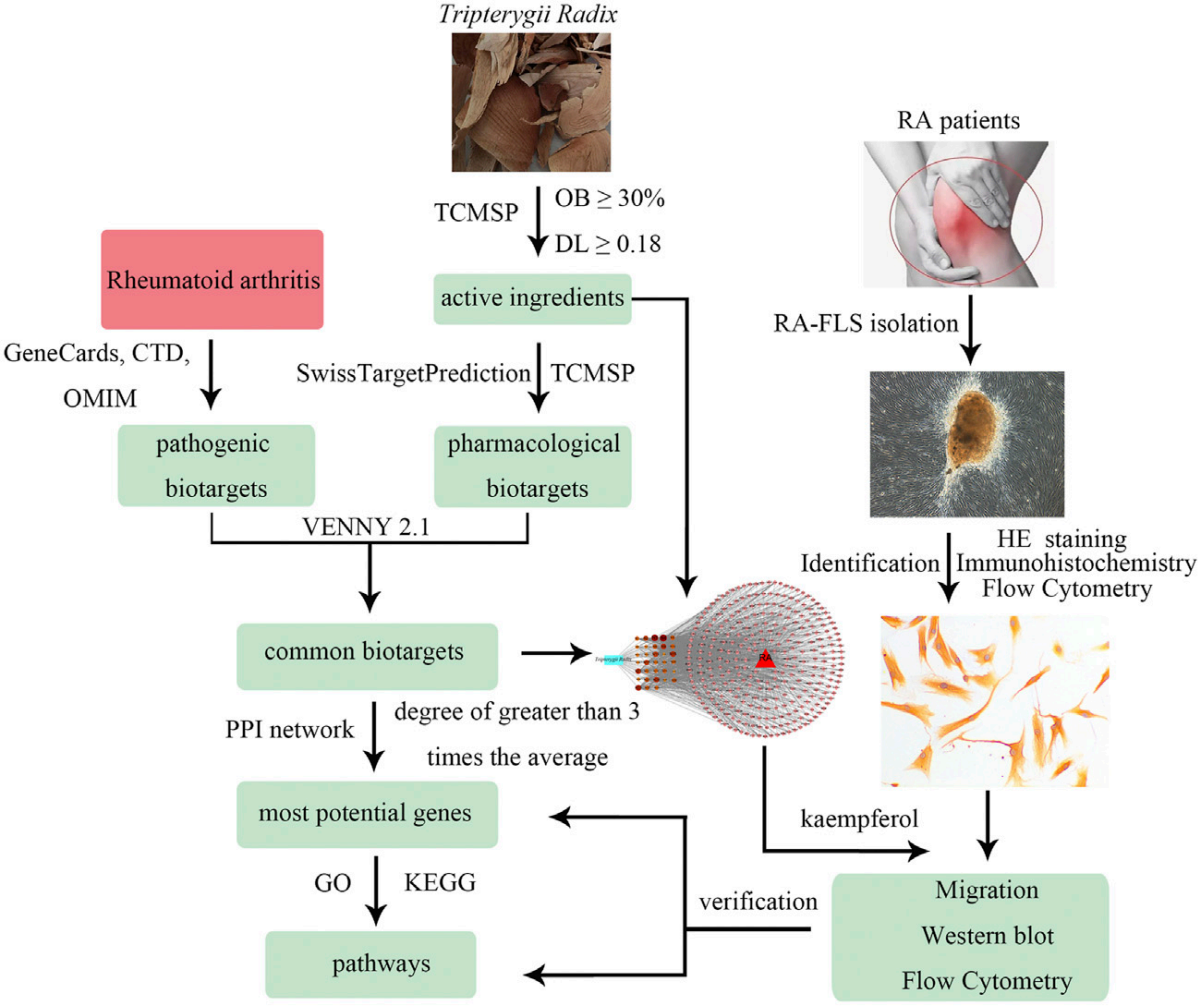
The workflow of this study.

Therefore, this report aims to predict the effective ingredients and the mechanism of action of *Tripterygii Radix* on RA based on network pharmacology. In addition, it aims to confirm the most effective compound and molecular targets through cell experiments. The study workflow is shown Figure 1.

## Materials and Methods

### Screening of Active Components and Pharmacological Biotargets of *Tripterygii Radix*

To determine the ingredients of *Tripterygii Radix*, ADME features were obtained from the Traditional Chinese Medicine Systems Pharmacology Database and Analysis Platform (TCMSP, https://tcmspw.com/) (Wan et al., 2019), a unique pharmacology platform designed for herbal medicine. In accordance with the pharmacokinetic characteristics in TCMSP database, the chemical components that satisfied both oral bioavailability (OB) ≥ 30% and drug-likeness (DL) ≥ 0.18 were selected as active components. The corresponding pharmacological biotargets were reaped (Huang et al., 2017). After inputting the selected active components into the SwissTargetPrediction database (http://www.swisstargetprediction.ch/) (Daina et al., 2019), the pharmacological biotargets were obtained. The final biotargets of active components were retained after removing duplicate targets of the results.

### Collecting Pathogenic Biotargets of RA

After deleting the duplicate targets, the information on pathogenic biotargets of RA was collected from the GeneCards database (GeneCards, https://genecards.weizmann.ac.il/v3/), Comparative Toxicogenomics Database (CTD, https://ctdbase.org/), and Online Mendelian Inheritance in Man (OMIM, https://www.omim.org/) (Rappaport et al., 2017; Davis et al., 2019).

### Choosing Common Biotargets and Construction of Protein-Protein Interaction (PPI) Network

VENNY 2.1 (http://bioinfogp.cnb.csic.es/tools/venny/) was used to obtain the common targets (Liang et al., 2019). The drug-compounds-biotargets-diseases network was visualized by Cytoscape 3.7.1 software. The nodes in the network represented active components and target genes, while the edges indicated the ingredients that interacted with the targets. The common genes were imported to the STRING database (https://string-db.org/) to construct the PPI network after deleting orphaned nodes. Then, the most potential targets were chosen on the basis of the degree value (Athanasios et al., 2017; Zhang et al., 2019).

### Gene Ontology and Kyoto Encyclopedia of Genes and Genomes Pathway Enrichment Analyses

According to the average degree values originating from PPI results, the most important target genes were selected. GO function analysis, including biological process (BP), cell component (CC), and molecular function (MF) terms, and the KEGG pathway analysis were carried out to predict the possible courses and pathways of treatment using the ClueGO plugin (Hua et al., 2019). The terms with values of *p* < 0.05 were considered as significant processes and pathways.

### RA-FLS Isolation

The RA-FLS cells was isolated and cultured by explant adherent culture method. The synovial tissue was obtained from three patients (2 male and 1 female; 0–70 years old) during joint replacement surgery at The Second Affiliated Hospital of Guizhou University of Traditional Chinese Medicine. All patients were diagnosed with active RA according to 2010ACR/EULAR Classification Criteria for Rheumatoid Arthritis and disease activity score in 28 joints and four variables, including C-reactive protein (DAS28-CRP) ≥ 3.2 (Fransen and Riel, 2009; Aletaha et al., 2010). The experiments were approved by the Medical Ethical Committee of The Second Affiliated Hospital of Guizhou University of Traditional Chinese Medicine (PY2019104), and all patients signed informed consent. The synovial tissues were washed five times with Phosphate-Buffered Saline (PBS, BI, Israel) supplemented with 2% penicillin/streptomycin (Gibco, United States). After removing irrelevant tissues, especially the adipose tissue, the synovial tissue was cut into one cubic centimeter pieces. All pieces were cultured to harvest RA-FLS in a flask with a complete medium containing Dulbecco’s Modified Eagle’s Medium (DMEM, Gibco, United States) supplemented with 20% fetal bovine serum (BI, Israel) and 1% penicillin/streptomycin (Gibco, United States) at 37°C and 5% CO_2_-enriched atmosphere. Culture media were replenished every 4 days, and cells were subcultured at a ratio of 1:2 when they reached 90% confluence. RA-FLS were used for experiments between third and sixth passage.

### Cell Identification

Hematoxylin and eosin (HE) staining and immunohistochemistry were used to identify RA-FLS. The third passage FLS were stained using a HE staining kit (Solarbio, China) and incubated with a rabbit anti-vimentin antibody (Abcam, United Kingdom), followed by probing with a horseradish peroxidase (HRP)-conjugated goat anti-rabbit IgG antibody (ZSGB, China) and developing with 3,3′-diaminobenzidine reagent (ZSGB, China). The FLS were counterstained with hematoxylin.

The flow cytometry was used to get the purity of RA-FLS staining for cell surface markers CD90 or VCAM-1. The third passage FLS (1 × 105/ml) suspended in 1 ml of PBS in 12 × 75 mm2 tubes were centrifuged at 200 g for 5 min at 4°C and the supernatant was removed. The cells were incubated 30 min after 5 μL of FITC anti-CD90 (Abcam, United Kingdom) or APC anti-VCAM-1 (Abcam, United Kingdom) was added in dark at 4°C. After wasded three times, the cells were resuspended in 500 µL of a buffer solution and analyzed by flow cytometer (BD, United States).

### RA-FLS Intervention

The third passage RA-FLS were pre-treated with complete medium, 0.2% dimethyl sulfoxide (DMSO; Solarbio, China), 25 μM kaempferol (Shanghai Winherb, China), or 50 μM lenalidomide (Abcam, United Kingdom) for 24 h. All reagents were dissolved in DMSO and diluted to working concentrations with complete medium.

### Migration Assays

The RA-FLS (1 × 105/ml) were pre-treated with different working concentrations of test agents for 24 h. One day prior to setting up the Transwell migration assay, the RA-FLS were serum-starved overnight. A total of 3 × 104 cells were resuspended in 100 μL serum-free medium and seeded onto the upper chamber of a transwell migration assay plate (8 μm pores, Corning, United States), while the lower chamber was filled with 800 μL of complete medium. After 24 h incubation, media within the transwell inserts were carefully removed. Cells were fixed with 1 ml 2% paraformaldehyde. Then. the cells settling on the upper chamber were carefully removed with a cotton swab, while those migrated to the lower surface were stained with HE. Cell count was performed under a microscope (Olympus, Japan).

### Western Blot Assays

The cell lysates containing 40 μg total protein were loaded onto 10% sodium dodecyl sulfate- polyacrylamide gel electrophoresis (SDS-PAGE) gels (Bio-Rad, United States). The separated protein bands were transferred onto nitrocellulose membranes. After blocking with 5% skim milk in Tris-buffered-saline-Tween (TBST) 1.5 h, membranes were incubated with different primary antibodies, including those against JUN (Abcam, United Kingdom; 1:1,000), P-JUN (phosphor-S100, Abcam, United Kingdom; 1:1,000), protein kinase B (AKT1; Abcam, United Kingdom; 1:10,000), P-AKT1 (phosphor-S473, Abcam, United Kingdom; 1:5,000), caspase-3 (CASP3; Abcam, United Kingdom; 1:500), tumor necrosis factor receptor 1 (TNFR1; Abcam, United Kingdom; 1:5,000), and TNFR2 (Abcam, United Kingdom; 1:10,000) for overnight at 4°C. The membranes were washed five times with TBST, then peroxidase-conjugated goat anti-rabbit IgG antibody (Pumei, China) was used as a secondary antibody to incubate for 1 h. After another washed five times with TBST, enhanced chemiluminescence substrate (Bio-Rad, United States) was used to detect the amount of target proteins. ImageJ software was employed to quantify the relative intensity following normalization with β-actin (Cell Signaling, United States; 1:1,000). All experiments were repeated thrice.

### Flow Cytometry Assays

Multiparameter flow cytometry permits the simultaneous detection of two or more cytokines. Multiparameter flow cytometry was applied to detect the concentrations of TNF-α and IL-6 from the cell supernatants pre-treated with different working concentrations of test agents. The supernatants were obtained after pre-treatment for 24 h. The tests were performed according to the manufacturer’s protocols (RAISECARE, China). In brief, 25 μL of a buffer solution, 25 μL of the studied sample or standard, 25 μL of capture microsphere antibody, and 25 μL of a detection antibody were consecutively added to each tube. The tubes were incubated and vibrated (400–500 rpm) at 20°C in the dark for 2 h. The tubes were treated with 25 μL streptavidin-phycoerythrin for 0.5 h, washed with 500 μL of a wash buffer, and centrifuged. The supernatant was discarded and the pellet was resuspended in 200 µL of a buffer solution and analyzed by flow cytometer (BD, United States).

### Statistical Analysis

Statistical analysis was performed using the SPSS 17 software package (SPSS Inc., United States). One-way analysis of variance was used to compare the means of different groups. A value of *p* < 0.05 was considered as statistically significant.

## Results

### Active Components of *Tripterygii Radix*

OB is a central pharmacokinetic parameter in the ADME process and is associated with the absorption and delivery of an orally administered drug. DL represents the druggability of a drug and serves as a qualitative alternative to study pharmacokinetic and pharmaceutical properties. A total of 144 chemical components were detected from *Tripterygii Radix*, of which only 33 were considered as active components because they satisfied the criteria of OB ≥ 30% and DL ≥ 0.18 (Table 1).

**TABLE 1:** Active components with OB ≥ 30% and DL ≥ 0.18.

No	Mol ID	Molecule name	OB (%)	DL	CAS
N1	MOL000211	Mairin	55.38	0.78	472-15-1
N2	MOL000296	Hederagenin	36.91	0.75	465-99-6
N3	MOL000358	Beta-sitosterol	36.91	0.75	83-46-5
N4	MOL000422	Kaempferol	41.88	0.24	520-18-3
N5	MOL000449	Stigmasterol	43.83	0.76	83-48-7
N6	MOL002058	Medioresinol	57.2	0.62	31008-18-1
N7	MOL003182	(+)-Medioresinol di-O-beta-D-glucopyranoside_qt	60.69	0.62	/
N8	MOL003184	Neotriptophenolide	45.42	0.53	81827-74-9
N9	MOL003185	(1R,4aR,10aS)-5-hydroxy-1-(hydroxymethyl)-7-isopropyl-8-methoxy-1,4a-dimethyl-4,9,10,10a-tetrahydro-3H-phenanthren-2-one	48.84	0.38	110187-23-0
N10	MOL003187	Triptolide	51.29	0.68	38748-32-2
N11	MOL003196	Tryptophenolide	48.5	0.44	74285-86-2
N12	MOL003199	5,8-Dihydroxy-7-(4-hydroxy-5-methyl-coumarin-3)-coumarin	61.85	0.54	125124-67-6
N13	MOL003208	Celafurine	72.94	0.44	/
N14	MOL003209	Celallocinnine	83.47	0.59	/
N15	MOL003210	Celapanine	30.18	0.82	52658-32-9
N16	MOL003211	Celaxanthin	47.37	0.58	472-74-2
N17	MOL003217	Isoxanthohumol	56.81	0.39	70872-29-6
N18	MOL003225	Hypodiolide A	76.13	0.49	139122-81-9
N19	MOL003229	Triptinin B	34.73	0.32	/
N20	MOL003231	Triptoditerpenic acid B	40.02	0.36	147362-43-4
N21	MOL003232	Triptofordin B1	39.55	0.84	/
N22	MOL003244	Triptonide	68.45	0.68	38647-11-9
N23	MOL003245	Triptonoditerpenic acid	42.56	0.39	139953-20-1
N24	MOL003248	Triptonoterpene	48.57	0.28	/
N25	MOL003266	21-Hydroxy-30-norhopan-22-one	34.11	0.77	/
N26	MOL003278	Salaspermic acid	32.19	0.63	71247-78-4
N27	MOL003280	Triptonolide	49.51	0.49	/
N28	MOL003283	Isolariciresinol	66.51	0.39	548-29-8
N29	MOL004443	Zhebeiresinol	58.72	0.19	151636-98-5
N30	MOL005828	Nobiletin	61.67	0.52	478-01-3
N31	MOL007415	Aurantiamide acetate	58.02	0.52	56121-42-7
N32	MOL007535	(5,8,9S,10R,13R,14S,17R)-17-[(1R,4R)-4-ethyl-1,5-dimethylhexyl]-10,13-dimethyl-2,4,5,7,8,9,11,12,14,15,16,17-dodecahydro-1H-cyclopenta [a]phenanthrene-3,6-dione	33.12	0.79	/
N33	MOL011169	Peroxyergosterol	44.39	0.82	2061-64-5

### Biotargets and PPI Network

**FIGURE 2:**
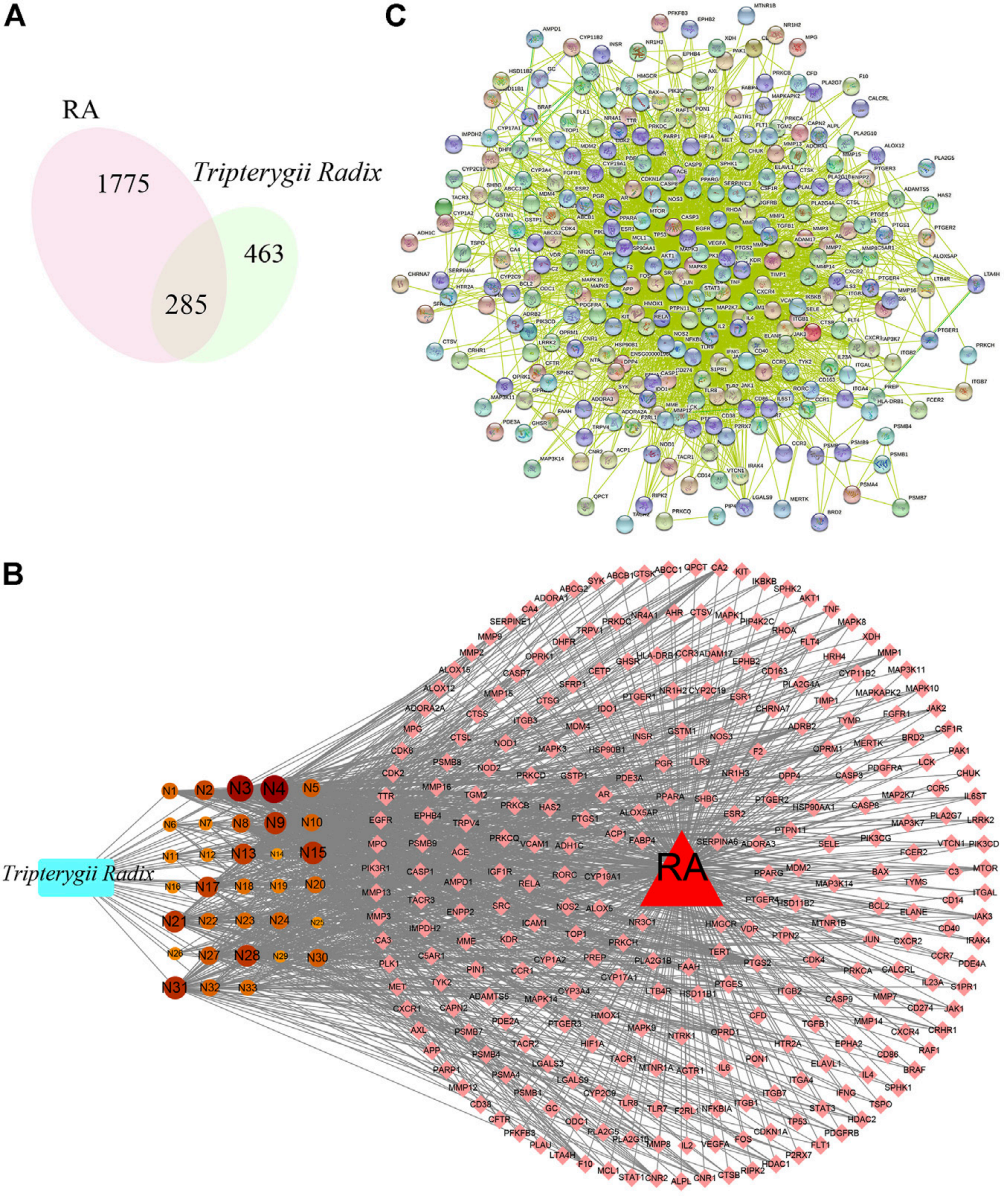
**(A)** represents the venn digram of biotargets between Tripterygii Radix and RA, While **(B)** represents the drug-active components-biotargets-disease network of Tripterygii Radix against RA. The rectangle represents Tripterygii Radix, the circle nodes represent active components, the diamond nodes represent biotargets, and the triangle represent RA. The 33 components nodes changed with the degree value. The higher the degree value, the bigger was the node. **(C)** represents the PPI network of the common biotargets.

A total of 748 pharmacological biotargets resulted from TCMSP and SwissTargetPrediction databases after ruling out non-human species. In total, 2060 pathogenic biotargets were selected from GeneCards, CTD, and OMIM, and 285 common biotargets were chosen by VENNY 2.1 **(** Figure 2A). The drug-compounds-biotargets-diseases network was visualized by Cytoscape 3.7.1 software, which revealed the effects of *Tripterygii Radix* on RA through the common biotargets (Figure 2B). The network contained 320 nodes and 1,304 edges. The average degree among the 33 active components was 30.84, while the top three compounds in conformity with the degree values were kaempferol, beta-sitosterol, and aurantiamide acetate that comprised the therapeutic constituents.

The PPI network (Figure 2C) constructed with 285 nodes and 3,741 edges for text-mining information and interaction predictions revealed 285 common biotargets. The average degree value of PPI network was 39.151, and 14 target genes (Table 2) were greater than three times the average degree value, indicative of them as the potential genes related to RA.

**TABLE 2:** Genes with greater than three times the average degree value.

No	Protein	Gene	Uniprot ID	Degree
1	Interleukin-6	*IL6*	P05231	184
2	Tumor necrosis factor	*TNF*	P01375	176
3	RAC-alpha serine/threonine-protein kinase	*AKT1*	P31749	169
4	Vascular endothelial growth factor A	*VEGFA*	P15692	162
5	Cellular tumor antigen p53	*TP53*	P04637	149
6	Mitogen-activated protein kinase 3	*MAPK3*	P27361	144
7	Signal transducer and activator of transcription 3	*STAT3*	P40763	134
8	Epidermal growth factor receptor	*EGFR*	P00533	133
9	Proto-oncogene tyrosine-protein kinase Src	*SRC*	P12931	132
10	Prostaglandin G/H synthase 2	*PTGS2*	P35354	126
11	Caspase-3	*CASP3*	P42574	125
12	Mitogen-activated protein kinase 1	*MAPK1*	P28482	124
13	Transcription factor AP-1	*JUN*	P05412	121
14	Mitogen-activated protein kinase 8	*MAPK8*	P45983	121

Abbreviation of the genes correspond to translated protein

### BP, CC, MF, and Pathways of *Tripterygii Radix* on RA

**FIGURE 3:**
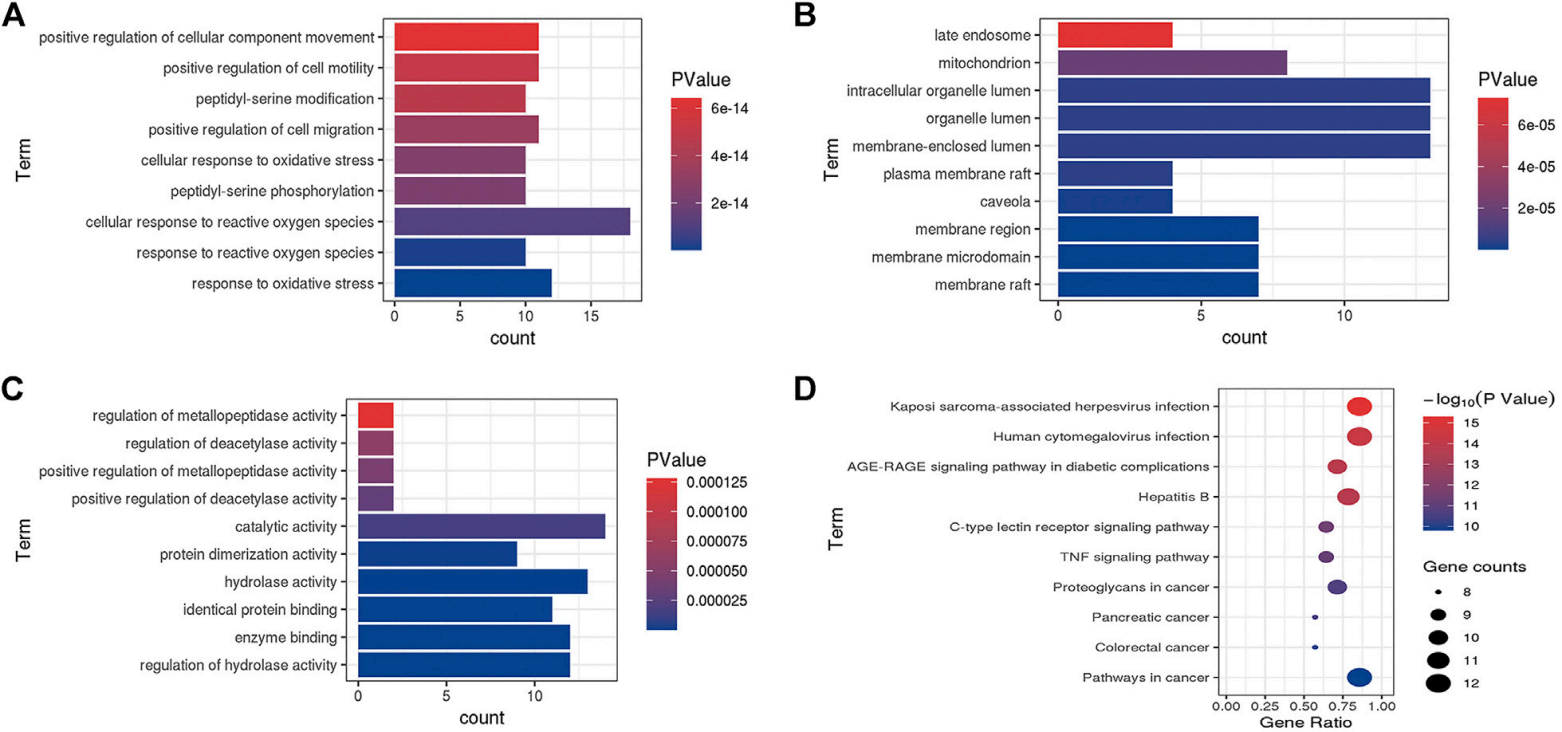
Go enriched terms of potential targets for biological processes **(A)**, cellular components **(B)** and molecular functions **(C)** and KEGG enriched terms **(D)**.

The GO function and KEGG pathway enrichment analyses were executed under the condition of *p* < 0.05 using the ClueGO plugin of Cytoscape 3.7.1. BP contained 659 terms, including response to oxidative stress, response to reactive oxygen species, and cellular response to reactive oxygen species. The top 10 terms are shown in Figure 3A in accordance with the lowest *p* value. CC only contained 11 terms such as membrane raft, membrane microdomain, and membrane region; the top 10 terms are shown in Figure 3B. In addition, the MF comprised 98 terms, including regulation of hydrolase activity, enzyme binding, and identical protein binding. The top 10 terms are shown in Figure 3C. KEGG pathway enrichment analysis indicated 104 terms such as AGE-RAGE signaling pathway in diabetic complications, C-type lectin receptor signaling pathway, and TNF signaling pathway; these along with the top 10 terms with lowest *p* values are shown in Figure 3D.

### HE Staining, Immunohistochemistry and Isolation Purity

**FIGURE 4:**
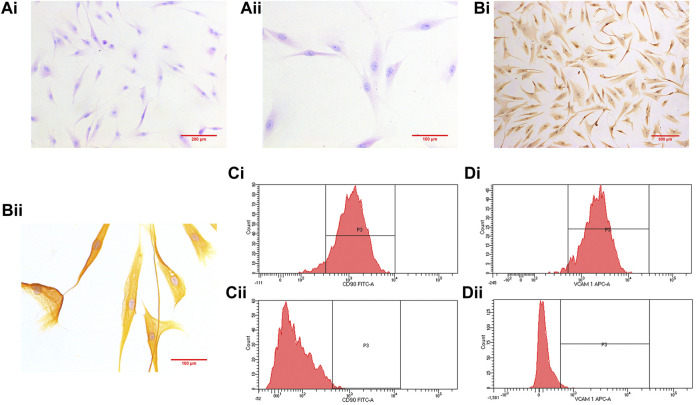
[**(A)**, i] (×100) and [**(A)**, ii] (×200) show HE staining results. Immunohistochemistry results are shown in [**(B)**, i] (×100) and [**(B)**, ii] (×400). [**(C)**, i] shows the purity of the isolation cells marked with FIDC anti- CD90 (96.6 ± 1.44%; *n* = 4), and [**(C)**, ii] represents control [**(D)**, i] shows the purity of the isolation cells marked with APC anti-VCAM-1 (97.1 ± 0.39%; *n* = 4), and [**(D)**, ii] represents control.

The third passage RA-FLS were subjected to HE staining and immunohistochemistry after being purified by passage culture. The cells were spindle shaped with blue nuclei and red cytoplasm (Figure 4A). The cytoplasm was brown with blue nuclei in immunohistochemistry, indicative of positive results (Figure 4B). Tissue origin, cell shape, and positive immunohistochemistry results demonstrated that the observed cells were RA-FLS. The purity of the isolation cells was 96.6 ± 1.44% (*n* = 4) marked with FITC anti-CD90 or 97.1 ± 0.39% (*n* = 4) APC anti-VCAM-1 respectively (Figures 4C,D).

### Effect of Kaempferol on RA-FLS Migration

**FIGURE 5:**
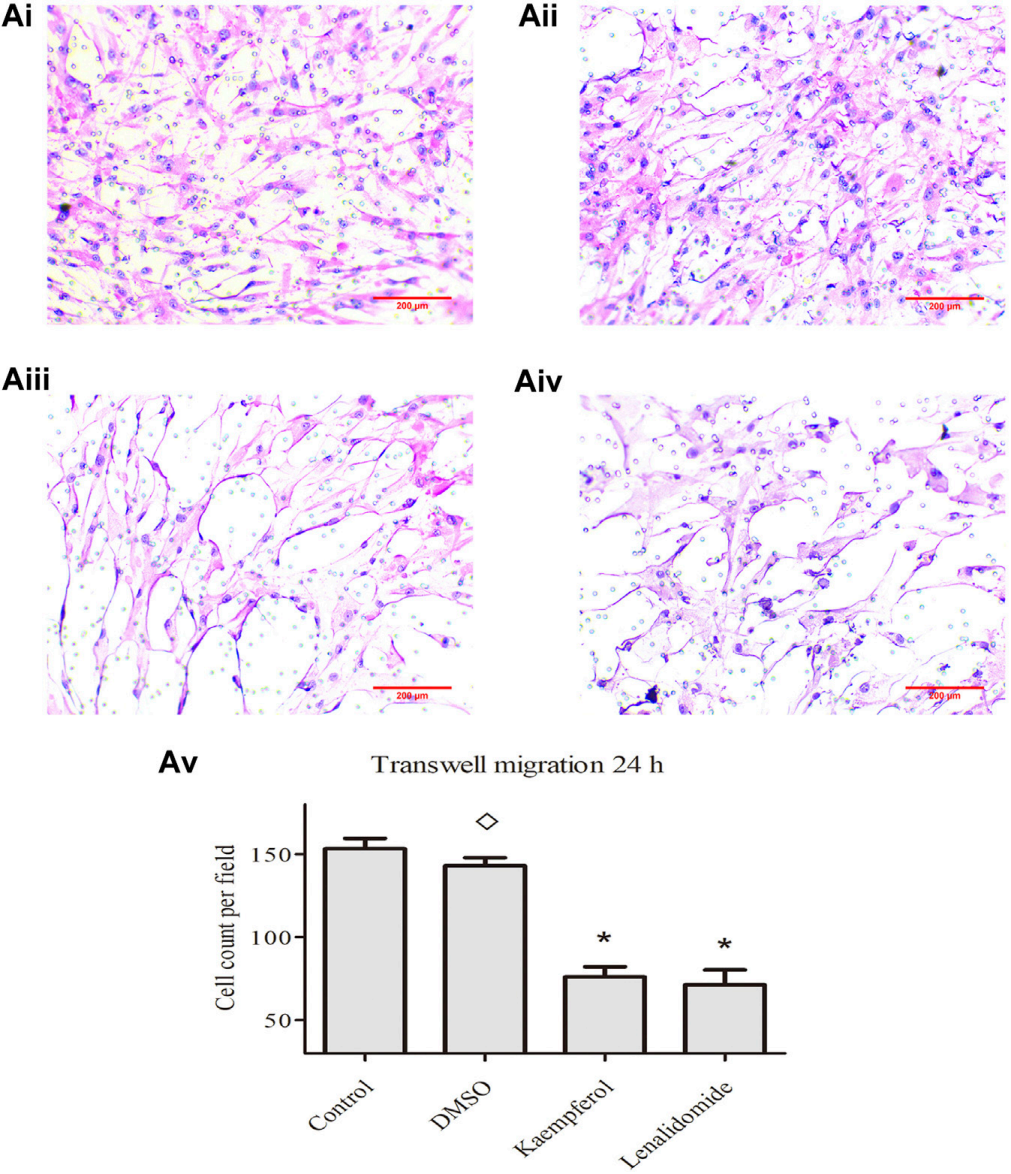
**(A)** i, ii, iii, and iv] show the effects of control, DMSO, kaempferol and lendidomide treatments on cell migration. Results in [**(A)**, v] are shown as means ± standard deviation (SD; *n* = 3), *p < 0.05, ^◊^ *p* > 0.05 vs. control treatement.

The effects of control, DMSO, kaempferol, and lenalidomide treatments on cell migration are shown in Figure 4, respectively. The images are captured under ×100 objective lens. In comparison with the control treatment, DMSO treatment had no effect on cell migration, but kaempferol (25 μM) and lenalidomide (50 μM) treatments inhibited cell migration (p < 0.05) (Figure 5).

### Impact of Kaempferol on Protein Expression of JUN, P-JUN, AKT1, P-AKT1, CASP3, TNFR1 and TNFR2

**FIGURE 6:**
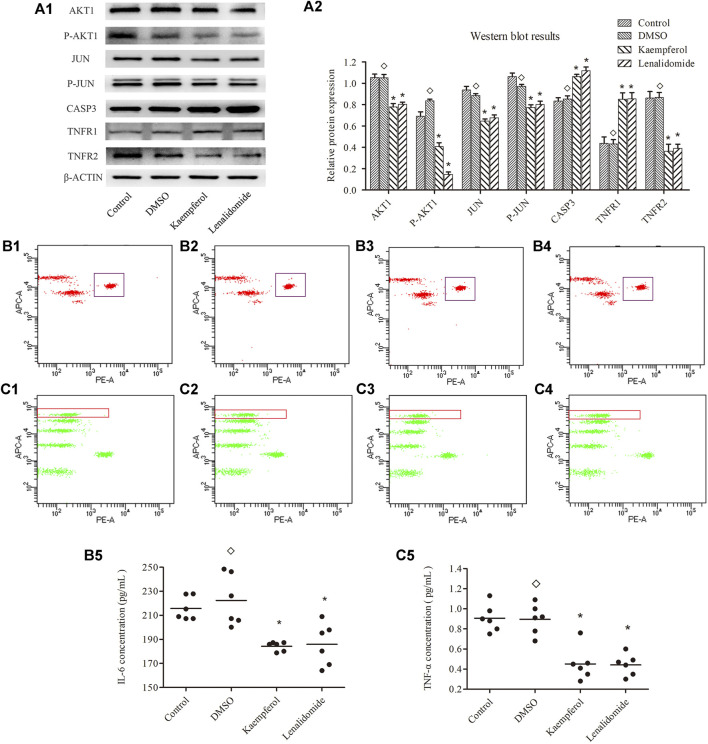
**(A)** shows the expression levels of JUN,P-JUN,AKTI,P-AKTI,CASP3, TNFR1 and TNFR2 in different groups (means ± SD; *n* = 3); *p* < 0.05, *p* > 0.05. as compard with the control group. **(B)** and **(C)** show the concentration of IL-6 and TNF-α, respectively.Ponits inside the rectangle represent IC-6 in B and TNF-α in C(B1, C1 represent contrpl group; B2, C2 represent DMSO grouop; B, C3 represent kaempferol group; B4, C4 represent lenalidomide group) Horizontal bars indicate meanns and *n* = 6; *p* < 0.05, *p* > 0.05 vs. control group.

As shown in Figure 6A, no significant differences were observed in the levels of these proteins between DMSO and control treatment groups (*p >* 0.05). Stimulation with kaempferol (25 μM) or lenalidomide (50 μM) resulted in the upregulation of the expression of CASP3 and TNFR1 as compared with the control treatment (p < 0.05) but downregulated JUN, P-JUN, AKT1, P-AKT1, and TNFR2 expression (p < 0.05).

### Regulation of Kaempferol on Cytokines of TNF-α and IL-6

Flow cytometry assay results are shown in Figures 6B,C. The cytokines levels of TNF-α and IL-6 were not significantly different between DMSO and control treatment groups (p < 0.05). However, the levels of TNF-α and IL-6 were lower in kaempferol (25 μM) and lenalidomide (50 μM) treatment groups than in the control group (p < 0.05).

## Discussion

Several clinical studies have proved *Tripterygii Radix* to be useful for RA treatment, but the underlying mechanism of action is unclear (Zhang et al., 2020). In this study, we predict the underlying mechanism of action by network pharmacology and further verified it with cell experiments. We screened 144 bioactive compounds from *Tripterygii Radix* depending on their OB and DL, and found kaempferol, beta-sitosterol, and aurantiamide acetate to be the most important active components, based on the degree values after the analysis of drug-compounds-biotargets-diseases network. Previous studies have shown all of these compounds to exert anti-inflammatory, anti-nociceptive, and immunomodulatory activities (Liu et al., 2015; Liu et al., 2019). In particular, kaempferol reduces the serum levels of IL-1β, IL-6, and TNF-α and inhibits cell proliferation through apoptosis and fibroblast growth factor receptor 3-ribosomal S6 kinase 2 (FGFR3-RSK2) signaling axis (Lee et al., 2018; Wang and Zhao, 2019).

Fourteen genes regarded as pivotal biological targets were shortlisted from the PPI network, including IL-6, TNF, AKT1, and JUN. IL-6 removes infectious agents and restore the damaged tissues through the activation of immune, hematological, and acute-phase responses (Tanaka et al., 2016). Human anti-IL-6 monoclonal antibody was shown to alleviate RA involving B cells and T cells, vascular endothelial growth factor (VEGF), and acute phase proteins (Narazaki et al., 2017). TNF induces apoptosis mediated by caspase activation and is highly responsive to osteoclasts in RA (Kiraz et al., 2016; McInnes and Schett, 2017). AKT1 regulates cell cycle and is associated with the downstream cellular mechanisms (Duggal et al., 2018). JUN influences apoptosis and macrophage activation in RA (Hannemann et al., 2017).

The outcomes of BP, CC, and MF included cellular response to reactive oxygen species, membrane microdomain, regulation of hydrolase activity, and others, indicating that it was a complex process related to organelles and cell membrane. KEGG pathway analysis indicated that AGE-RAGE signaling pathway in diabetic complications, C-type lectin receptor signaling pathway, and TNF signaling pathway were crucial signaling pathways related to RA treatment with the lowest *p* values. TNF signaling pathway mediates inflammatory responses and cell proliferation, differentiation, and death through the NF-κB and mitogen-activated protein kinase pathways (Borghi et al., 2019). TNFR1 and TNFR2 are two distinct receptors that accept exogenous signals in TNF signaling pathway (Jarosz-Griffiths et al., 2019). They both activate, either directly or indirectly, NF-κB and MAPKs (Noack and Miossec, 2017).

Therefore, TNF signaling pathway was thought to be one of the most latent treatment pathways, and some of the 14 biotargets (AKT1, JUN, CASP3, TNFR1, TNFR2, IL-6, TNF-α) were considered as potential target genes. Kaempferol was associated with the maximum number of potential target genes. Thus, kaempferol was deemed as the most valuable active component of Tripterygii Radix in RA, wherein it acts on the TNF-α signaling pathway. The cells from RA patients were verified to be RA-FLS. Kaempferol could inhibit RA-FLS migration and regulate the expression of AKT1, P-AKT1, JUN, P-JUN, CASP3, TNFR1, and TNFR2 proteins. The concentrations of IL-6 and TNF-α were reduced after intervention with kaempferol.

Kaempferol promoted TNFR1 expression, but inhibited TNFR2 expression. TNFR1’s cytoplasmic tail contains a death domain (DD), thereby allowing it to recruit the TNFR1-associated DD (TRADD); TNFR2, on the other hand, does not have an intracellular DD and recruits the TNFR associated factor (TRAF) 1 and 2 proteins instead (Holbrooket et al., 2019). Cell death can be initiated by TNFR1 through apoptosis (Zhu et al., 2019). TNFR2 interacts directly with TRAF1 or two promote cell survival signaling through NF-κB, MAPK, and Akt, promoting cell proliferation and tissue regeneration (Borghi et al., 2019).

In conclusion, kaempferol was found to be the most important active compound of *Tripterygii Radix* effective against RA. It acts through the TNF signaling pathway by modulating the expression of several biotargets (AKT1, JUN, CASP3, TNFR1, TNFR2, IL-6, TNF-α). Experiments with RA-FLS confirmed this effect. Further studies need to explore the therapeutic mechanism underlying *Tripterygii Radix* effects in RA.

## Data Availability

The datasets presented in this study can be found in online repositories. The names of the repository/repositories and accession number(s) can be found in the article/Supplementary Material.
