# Supplementary material for: Prediction and Verification of the Major Ingredients and Molecular Targets of Tripterygii Radix Against Rheumatoid Arthritis
Source: Front Pharmacol. 2021 Jun 8;12:639382. doi: 10.3389/fphar.2021.639382 (PMC8217827; doi:10.3389/fphar.2021.639382)
Supplement: Supplementary file 1 [file DataSheet2.PDF]

附件 6：

贵州中医药大学第二附属医院受试者知情同意书

试验项目名称：金乌健骨胶囊对类风湿关节炎滑膜细胞焦亡的影响	
研究单位：贵州中医药大学第二附属医院	电话：0851-85285555
研究负责人：马武开	职称：教授
紧急联系人：凌益	紧急联系电话： 13618582147

自愿受试者姓名：黄仁[illegible]　　病历号：200[illegible]417

性别：男　　年龄：59岁

通讯地址：中国贵州 大方县 [illegible]

电话：15761639428

(一)试验目的：

我们敬邀您参加本院有 3 位病人参加的医学研究计划。通过借助 RA 患者术后取下的滑膜组织，进行培养建立 RA 患者滑膜细胞模型，明确金乌健骨胶囊对类风湿关节炎滑膜细胞焦亡的影响机制。本实验将要对您的滑膜组织进行培养，特告知您相关实验事项，希望能得到您的理解与支持。

(二)试验方法：

（1）受试者标准及数目

- 受试者必须符合以下所有条件方能参加本试验
  - a. 活动期 RA 患者，DAS28>3.2；
  - b. 年龄区间在 18-70 岁的男性和（或）女性患者；
  - c. 近 3 个月未使用抗风湿药；
  - d. 不伴有其他风湿病，如系统性红斑狼疮、干燥综合征、严重的膝骨关节炎等。
- 下列所述通常都会列入纳入条件中最后一项说明：
  所有受试者或其监护人必须在进入试验前签署受试者同意书。
- 若有下列任何情况者，不能参加本试验：
  - a. 不符合纳入标准者；
  - b. 合并各种急慢性感染性或伴有传染性疾病，如结核、乙肝等者；
  - c. 合并严重疾病，如心、脑、肝、肾或造血系统等危重症者；
  - d. 妊娠或哺乳期妇女，精神病患者；
  - e. 研究者的判断，不宜入选者。

（2）试验设计及进行步骤

如您同意参加本研究，实验主持医师会先帮您做评估，以确认您是否合乎试验纳入条件。

（3）试验期限及进度

本试验将于2019年09月至2020年09月间进行，预估将有3位病人参与。

（4）评估及统计方法

采用SPSS20.0软件分析，计量资料以均数±标准差（ $\bar{x}$±s）表示，对符合正态分布满足方差齐性采用t检验、方差分析，不符合的采用非参数（秩和）检验；计数资料采用校正卡方检验、Fisher精确检验等；等级资料采用wilcoxon秩和检验分析。所有的统计检验均采用双侧检验，P<0.05为所检验的判别有统计意义。

（三）参与试验费用说明：

您参与本试验将不需额外支付任何费用。

(四)参与试验可能获得之效益：

本课题在前期临床和实验研究的基础上，使患者通过提供类风湿关节炎滑膜组织参与科学研究，探讨苗药金乌健骨方对类风湿关节炎滑膜细胞的调控机制，为开发民族药奠定实验及理论基础,为广大的类风湿关节炎患者提供一“简、便、效、廉”的苗医药方。

（五）可能产生之副作用及危险：

1.本研究为无害性研究，药物为治疗性药物，研究借助细胞培养方法进行，对手术患者及社会均不会造成其他特殊危害。

2.研究中滑膜组织获取需进行滑膜组织切除，有术后出血、感染、药物过敏等手术风险，但与其他常规滑膜切除术无差异，故相关预案同滑膜切除术。

3.对受试者在研究中可能承受的风险等不良事件的预防和应对预案详见相关附件。

(六)目前其它可能之疗法及其说明：

（七）您的权利和责任：

参加本临床试验您的个人权益将受以下条件保护：

若执行系依照所订试验计划书引起之伤害时，试验委托者将依法负损害赔偿责任。

1. 本临床试验计划之执行机构（本试验计划之药物已在我国上市使用）将维护您在试验过程当中应得之权益。

2. 您的隐私保护

(1) 研究医师及人员会保密您的医疗纪录，所收集到的数据、检查结果及医师诊断都会被保密，且会有一编码来保护您的姓名不被公开。除了有关机构依法调查外，我们会维护您的隐私。

(2) 试验所得数据可因学术性需要而发表，但对您之隐私（如姓名、病历号码...等）将不会公布，予绝对保密。

3. 若您在试验期间受到任何伤害或对您的权益产生疑问，请与＿凌益＿医师联系，其联系电话为＿13618582147＿。

（八）您无须提出任何理由，有拒绝参加试验之权利，可随时撤回同意退出实验，而此决定并不会引起任何不愉快或影响日后医师对您的医疗照护。

项目负责人签名：[signature]　　日期：2020.03.04

（九）本人已详阅上列各项资料，有关本临床试验计划之疑问也经试验主持人详细予以解释，了解整个实验的状况，并经过充份的考虑后，本人同意接受为此次临床试验之自愿受试验者。

自愿受试验者签名（或法定代理人）：黄仁静　　日期：2020.03.04

身份证号码：522422196004154615　　电话：15761639428
