# Supplementary material for: Prediction and Verification of the Major Ingredients and Molecular Targets of Tripterygii Radix Against Rheumatoid Arthritis
Source: Front Pharmacol. 2021 Jun 8;12:639382. doi: 10.3389/fphar.2021.639382 (PMC8217827; doi:10.3389/fphar.2021.639382)
Supplement: Supplementary file 2 [file DataSheet4.PDF]

Informed consent of subjects in the Second Affiliated Hospital of Guizhou University of Traditional Chinese Medicine

<table>
<tr><td colspan="2">Project name： Effect of Jinwujiangu Capsule on pyroptosis of Synovial Cells in Rheumatoid Arthritis</td></tr>
<tr><td>Research Institute: Second Affiliated Hospital of Guizhou University of Traditional Chinese Medicine</td><td>Tel: 0851-85285555</td></tr>
<tr><td>Research leader: WuKai Ma</td><td>Professor</td></tr>
<tr><td>Emergency contact:Yi Ling</td><td>Emergency contact number: 13618582147</td></tr>
<tr><td colspan="2">Volunteer name： Medical record number：
Gender： AGE：
Address：
Telephone:</td></tr>
<tr><td colspan="2">1. Purpose: We sincerely invite you to participate in the medical research program with three patients in our hospital. The synovial cell model of RA patients was established by culturing synovial tissue originated from RA patients after operation, and the effect of Jinwujiangu on pyroptosis of synovial cells in rheumatoid arthritis was clarified. In this experiment, we will cultivate your synovial tissue. We would like to inform you of the relevant experimental items to get your understanding and support.</td></tr>
<tr><td colspan="2">2.Method :
(1).The standard and number of subjects Subjects must meet all the following conditions before they can participate in this test
A.active RA patients, das 28 > 3.2;
B.male and/or female patients aged between 18 and 70;
C.no anti-rheumatic drugs have been used in recent 3 months;
D.without other rheumatism, such as systemic lupus erythematosus, Sjogren's syndrome and severe knee osteoarthritis.
The following statements are usually included in the last statement of inclusion conditions: All subjects or their guardians must sign the subject consent form before entering the trial. In case of any of the following circumstances, you cannot participate in this test:
A. those who do not meet the inclusion criteria;
B.those who are complicated with various acute and chronic infectious diseases, such as tuberculosis and hepatitis B;
C.patients with serious diseases, such as heart, brain, liver, kidney or hematopoietic system;
D. pregnant or lactating women, mental patients;
E. the judgment of the researcher is not suitable for selection.
(2) Test design and implementation steps
If you agree to participate in this study, the trial host doctor will help you to make an</td></tr>
</table>

assessment to confirm whether you meet the conditions for trial inclusion.

(3).Period and priogress

This trial will be conducted from September, 2019 to December, 2021. It is estimated that three patients will participate.

(4).Evaluation and statistical methods

SPSS20.0 software was used for analysis, and the measurement data were expressed as mean standard deviation ($\bar{x} \pm s$). The test of T and analysis of variance were used for the homogeneity of variance in accordance with normal distribution, and the nonparametric test (rank sum) was used for non-conformance. Corrected chi-square test and Fisher accurate test were used for counting data. Wilcoxon rank sum test was used to analyze the grade data. All statistical tests were conducted by bilateral tests, and $P < 0.05$ meant that the test was statistically significant.

3.Description of the cost of participating in the test:

There will be no extra cost for you to participate in this experiment.

4.Possible benefits from participating in the experiment:

On the basis of previous clinical and experimental research, this topic enables patients to participate in scientific research by providing synovial tissue of rheumatoid arthritis, and discusses the influence of Miao medicine Jinwujiangu prescription on the scorched death of synovial cells of rheumatoid arthritis, which lays an experimental and theoretical foundation for developing national medicine and provides a simple, convenient, effective and inexpensive Miao medicine prescription for the majority of rheumatoid arthritis.

5.Possible side effects and risks:

(1) This study is harmless, and the drugs are therapeutic drugs. The study is carried out by means of cell culture, which will not cause any other special harm to patients undergoing surgery and society.

(2) Synovial tissue resection is needed to obtain synovial tissue in the study. There are surgical risks such as postoperative bleeding, infection and drug allergy, but there is no difference with other conventional synovectomy, so the relevant plan is the same as synovectomy.

(3) Please refer to the relevant annexes for the prevention and response plan of adverse events such as risks that subjects may bear during the study.

6.Other possible therapies at present and their explanations:

7.Your rights and responsibilities:

Your personal rights and interests in this clinical trial will be protected by the following conditions: If the execution is in accordance with the test plan, the test client will be liable for damages according to law.

(1).The executing agency of this clinical trial plan (the drugs of this trial plan have been put on the market in China) will maintain your trial Rights and interests due in the process.

(2).Your privacy protection (1) The research doctors and personnel will keep your medical

records confidential, and the collected data, examination results and doctor's diagnosis will be kept confidential, and there will be a code to protect your name from being made public. We will protect your privacy, except for the investigation conducted by relevant agencies according to law.

(3).The data obtained from the experiment can be published for academic needs, but it is important for your privacy (such as name, medical record number, etc.) Will not be released and will be kept strictly confidential.

(4).If you suffer any injury during the trial or have questions about your rights and interests, please contact Dr. Yi Ling, its contact number is 13618582147.

8.You have the right to refuse to participate in the experiment without giving any reason, and you can withdraw your consent to withdraw from the experiment at any time, and this decision will not cause any unhappiness or affect your medical care in the future.

Signature of project leader: Date:

9.I have read all the above information carefully, and the questions about this clinical trial plan have been explained in detail by the trial host, and I have understood the whole experimental situation. After full consideration, I agree to accept this clinical trial as a voluntary subject.

Signature of voluntary subjects (or legal representative): Date:

ID card number: Telephone number:
