# Supplementary material for: Prediction and Verification of the Major Ingredients and Molecular Targets of Tripterygii Radix Against Rheumatoid Arthritis
Source: Front Pharmacol. 2021 Jun 8;12:639382. doi: 10.3389/fphar.2021.639382 (PMC8217827; doi:10.3389/fphar.2021.639382)
Supplement: Supplementary file 4 [file DataSheet3.PDF]

Informed consent of subjects in the Second Affiliated Hospital of Guizhou University of Traditional Chinese Medicine

<table>
<tr><td>Project name</td><td colspan="5">Effect of Jinwujiangu Capsule on pyroptosis of Synovial Cells in Rheumatoid Arthritis</td></tr>
<tr><td>Ethical approval number</td><td colspan="3">PY2019104</td><td>Start and end time of project</td><td>2018.01-2021.12</td></tr>
<tr><td>Item category</td><td colspan="5">Clinical trials of drugs</td></tr>
<tr><td rowspan="2">Research leader</td><td rowspan="2">WuKai Ma</td><td rowspan="2">academic/ educational background</td><td rowspan="2">doctoral candidate</td><td rowspan="2">Telephone number
e-mail</td><td>13608553702</td></tr>
<tr><td>walker55@163.com</td></tr>
<tr><td>research contents</td><td colspan="5">Synovial fibroblast-like cell were obtained fron RA patients. By establishing RA-FLS model of RA patients, we observed the reffect of serum containing Jinwujiangu Capsule on pyroptosis of RA-FLS and its mechanism in the pathogenesis of RA bone destruction. MTT assay, scratch test and Transwell test were used to detect the inhibitory effect of serum containing Jinwujiangu capsule on RA-FLS. Western blot (WB) was used to study the effect of Wujiangu capsule rabbit serum on the activities of NLRP3, Caspase-1/3/4/5/11, GasderminD, ASC and HMGB1 protein in RA-FLS of RA patients. Indirect detection of cell membrane permeability by LDH release; Membrane permeability was observed by AO/EB and Hoechst/PI staining, and cell morphology was observed by fluorescence microscope. The effect of serum containing wujiangu capsule on IL-1β, IL-18 and Caspase-1/3 was studied by ELISA.</td></tr>
</table>

main researcher commit：

The above contents are true. If approved, ______will study in strict accordance with the scheme provided, abide by the relevant regulations of the hospital ethics committee, and accept the supervision of the ethics committee.

Date：

Examination and approval opinions of hospital ethics Committee:

After reviewing this research project, the researcher's qualification meets the research requirements, the research design and method are reasonable, there are risk prevention and response measures, and the principles of ethical review are met, so it is agreed to carry out this research.

Signature and seal of Chairman:

Medical Ethics Committee of the Second Affiliated Hospital of Guizhou University of Traditional Chinese Medicine

Date:
